# Regional distribution of mechanical strain and macrophage-associated lung inflammation after ventilator-induced lung injury: an experimental study

**DOI:** 10.1186/s40635-024-00663-2

**Published:** 2024-09-03

**Authors:** Francesco Liggieri, Elena Chiodaroli, Mariangela Pellegrini, Emmi Puuvuori, Jonathan Sigfridsson, Irina Velikyan, Davide Chiumello, Lorenzo Ball, Paolo Pelosi, Sebastiano Stramaglia, Gunnar Antoni, Olof Eriksson, Gaetano Perchiazzi

**Affiliations:** 1 The Hedenstierna Laboratory, Department of Surgical Sciences, Uppsala University, Akademiska Sjukhuset-Ing. 40, Tr. 3, 75185 Uppsala, Sweden; 2 Dipartimento di Scienze Diagnostiche e Chirurgiche Integrate, Università di Genova, Genoa, Italy; 3 Department of Anesthesia and Intensive Care, ASST Santi Paolo e Carlo, San Paolo University Hospital, Milan, Italy; 4 Science for Life Laboratory, Department of Medicinal Chemistry, Uppsala University, Uppsala, Sweden; 5 Department of Anesthesia and Intensive Care Medicine, Uppsala University Hospital, Uppsala, Sweden; 6 PET Center, Center for Medical Imaging, Uppsala University Hospital, Uppsala, Sweden; 7 Department of Health Sciences, University of Milan, Milan, Italy; 8 Coordinated Research Center on Respiratory Failure, University of Milan, Milan, Italy; 9 Department of Physics, National Institute for Nuclear Physics, University of Bari Aldo Moro, Bari, Italy

## Abstract

**Background:**

Alveolar macrophages activation to the pro-inflammatory phenotype M1 is pivotal in the pathophysiology of Ventilator-Induced Lung Injury (VILI). Increased lung strain is a known determinant of VILI, but a direct correspondence between regional lung strain and macrophagic activation remains unestablished. [^68^Ga]Ga-DOTA-TATE is a Positron Emission Tomography (PET) radiopharmaceutical with a high affinity for somatostatin receptor subtype 2 (SSTR2), which is overexpressed by pro-inflammatory-activated macrophages. Aim of the study was to determine, in a porcine model of VILI, whether mechanical strain correlates topographically with distribution of activated macrophages detected by [^68^Ga]Ga-DOTA-TATE uptake.

**Methods:**

Seven anesthetized pigs underwent VILI, while three served as control. Lung CT scans were acquired at incremental tidal volumes, simultaneously recording lung mechanics. [^68^Ga]Ga-DOTA-TATE was administered, followed by dynamic PET scans. Custom MatLab scripts generated voxel-by-voxel gas volume and strain maps from CT slices at para-diaphragmatic (Para-D) and mid-thoracic (Mid-T) levels. Analysis of regional Voxel-associated Normal Strain (VoStrain) and [^68^Ga]Ga-DOTA-TATE uptake was performed and a measure of the statistical correlation between these two variables was quantified using the linear mutual information (LMI) method.

**Results:**

Compared to controls, the VILI group exhibited statistically significant higher VoStrain and Standardized Uptake Value Ratios (SUVR) both at Para-D and Mid-T levels. Both VoStrain and SUVR increased along the gravitational axis with an increment described by statistically different regression lines between VILI and healthy controls and reaching the peak in the dependent regions of the lung (for strain in VILI vs. control was at Para-D: 760 ± 210 vs. 449 ± 106; at Mid-T level 497 ± 373 vs. 193 ± 160; for SUVR, in VILI vs. control was at Para-D: 2.2 ± 1.3 vs. 1.3 ± 0.1; at Mid-T level 1.3 ± 1.0 vs. 0.6 ± 0.03). LMI in both Para-D and Mid-T was statistically significantly higher in VILI than in controls.

**Conclusions:**

In this porcine model of VILI, we found a topographical correlation between lung strain and [^68^Ga]Ga-DOTA-TATE uptake at voxel level, suggesting that mechanical alteration and specific activation of inflammatory cells are strongly linked in VILI. This study represents the first voxel-by-voxel examination of this relationship in a multi-modal imaging analysis.

## Introduction

In 1744 for the first time, it was observed that mechanical forces generated by uncontrolled manual ventilation could lead to injury and biological damage [1]. However, it is necessary to arrive at the implementation of mechanical ventilation on a routine basis to hypothesize the role of barotrauma as a complication [2]. Later, the focus moved from the high pressures exerted against the alveolar walls to the volumes that the lungs had to bear [3]. Still, it was necessary to come to the 2000s to convey several laboratory observations [4] into a unifying clinical hypothesis [5]. This, in turn led to two different research paths: the attempt to define safe limits of strain during mechanical ventilation [6] and the effort of measuring the elastic forces on a regional basis using image analysis of computer tomograms [7].

Different studies have reported increased inflammatory markers in animal models exposed to excessive lung strain during mechanical ventilation [8, 9]. However, these temporal associations never arrived to demonstrate the presence of inflammatory cells where a numerically estimated local strain of the lung was high: the studies of Borges and coworkers, based on Positron emission tomography (PET) with 2-deoxy-2-[^18^F]fluoro-d-glucose ([^18^F]FDG), although observed a concentration of the marker in specific areas of the lung, did not compute the local strain of these [10, 11].

A possible further complication has been the lack of a specific PET tracer for the cells intervening in the inflammatory process in the lung. The mentioned [^18^F]FDG is a glucose analog that labels all the cells using a glycolytic pathway: for these reasons, it requires a sophisticated data treatment for attenuating the effects of multicompartmental kinetics of the substance [12, 13]. A step forward has been performed by Bitker et al. [14, 15] using the tracer [*N*-methyl-^11^C]-1-(2-chlorophenyl)-*N*-(1-methylpropyl)-3-isoquinoline-carboxamide (from here on shortened as [^11^C]PK11195) which can mark the presence of macrophages. However, the generic presence of macrophages does not warrant the existence of an ongoing inflammatory process.

In fact, macrophages responding to environmental stimuli undergo a process of differentiation known as macrophagic polarization which results in a spectrum of diversely differentiated phenotypes, between the classically activated (M1) and alternatively activated (M2) macrophages. The M1 phenotype plays a pro-inflammatory role, producing cytokines that promote the acute phase of the inflammatory cascade, while the M2 macrophages play predominantly an immunoregulatory role, being involved in later stages of inflammation [16].

The discovery that activated, proinflammatory macrophages in phase M1 express on their surface the somatostatin receptors type 2 (SSTR2) [17] opened new perspectives in this field of research. The clinically available PET-tracer [^68^Ga] Ga-DOTA-TATE can avidly bind with high affinity to SSTR2 [18] and constitute a tool to analyze the spatial distribution of the inflammatory process related to lung injury at voxel level, as recently demonstrated by Puuvuori et al. [19].

We profited by a combined scanner performing positron emission tomography and computed tomography (PET–CT), using [^68^Ga]Ga-DOTA-TATE for activated macrophages and a computation method to estimate lung strain at the voxel level, to reveal whether activated macrophages are distributed in lung regions where high strain is located.

We hypothesized that high mechanical strain is topographically associated with the activation of the inflammatory cascade, as mirrored by the presence of polarized M1 macrophages. To assess this hypothesis, one group of animals experienced a dual-hit lung injury and was compared with a group of healthy controls. Both groups were ventilated using the same strategy, except for the limited duration of injurious ventilation in the VILI group. We analyzed the strain distribution to which the animal was exposed during injurious mechanical ventilation. Being computed tomography and positron emission tomography different modes of imaging, this purpose was performed using analysis methods able to observe whether information portrayed by one imaging mode was mirrored by the other.

## Methods

**Fig. 1:**
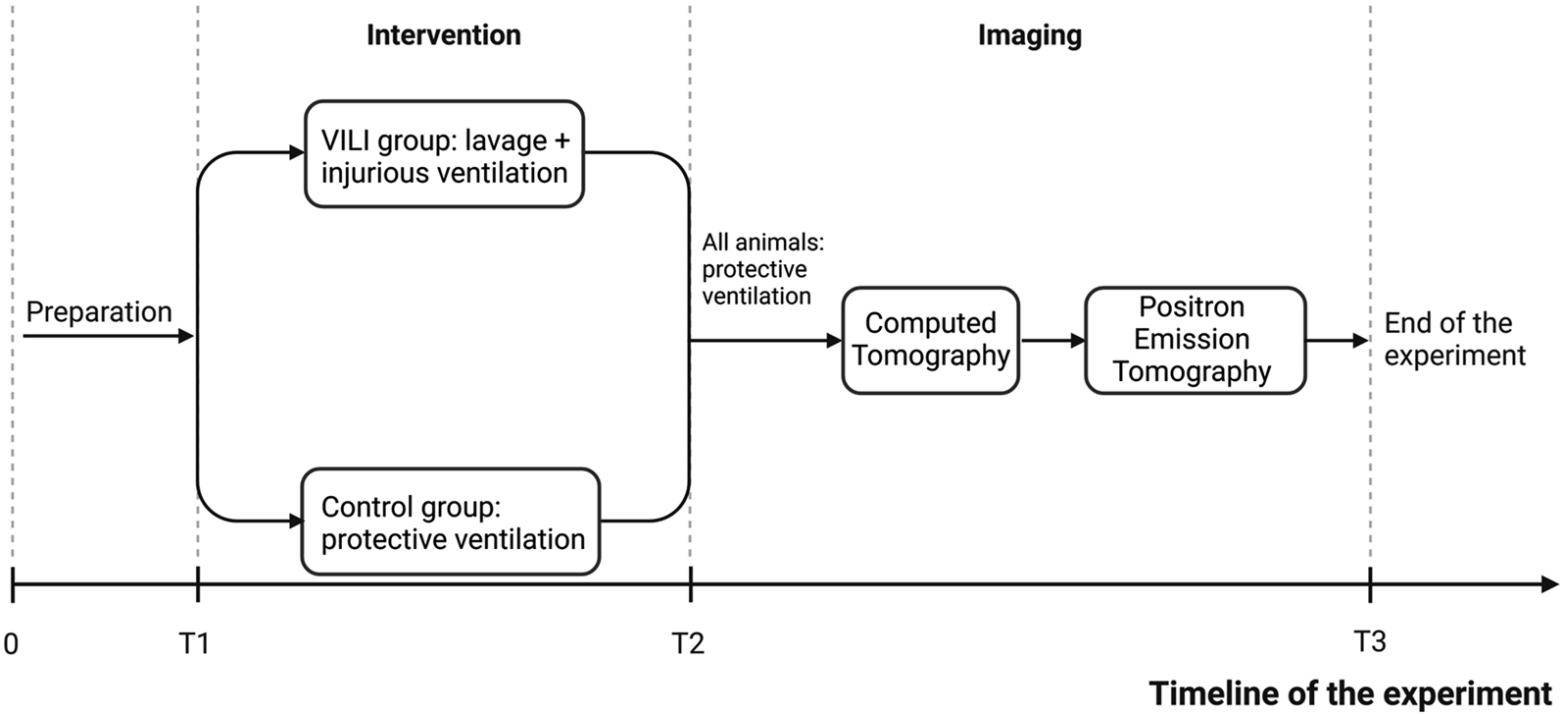
Timeline of the experiment. After the arrival at the laboratory and the surgical preparation, one group of animals underwent the induction of lung injury by repeated lung lavages and injurious ventilation, while the healthy controls were ventilated with a tidal volume of 6 ml/kg and PEEP adjusted according to the ARDSnet F_I_O_2_/PEEP table (in the graph labeled as “protective ventilation”: see details and references in the main text). After this phase, for the remaining part of the experiment, all the animals were ventilated with 6 ml/kg and PEEP according to the ARDSnet table. T1, T2, and T3 denote the timing for the coordinated measurement of arterial blood gas analysis and hemodynamic parameters. T1 corresponds to baseline after animal preparation, T2 after VILI induction, and T3 just before the piglet was euthanized. Please note that the total time of ventilation in the two groups was the same: each animal arrived at the laboratory at the same time of the day and reached the PET facility at a fixed time in the afternoon. (Graph created with BioRender.com)

**Fig. 2:**
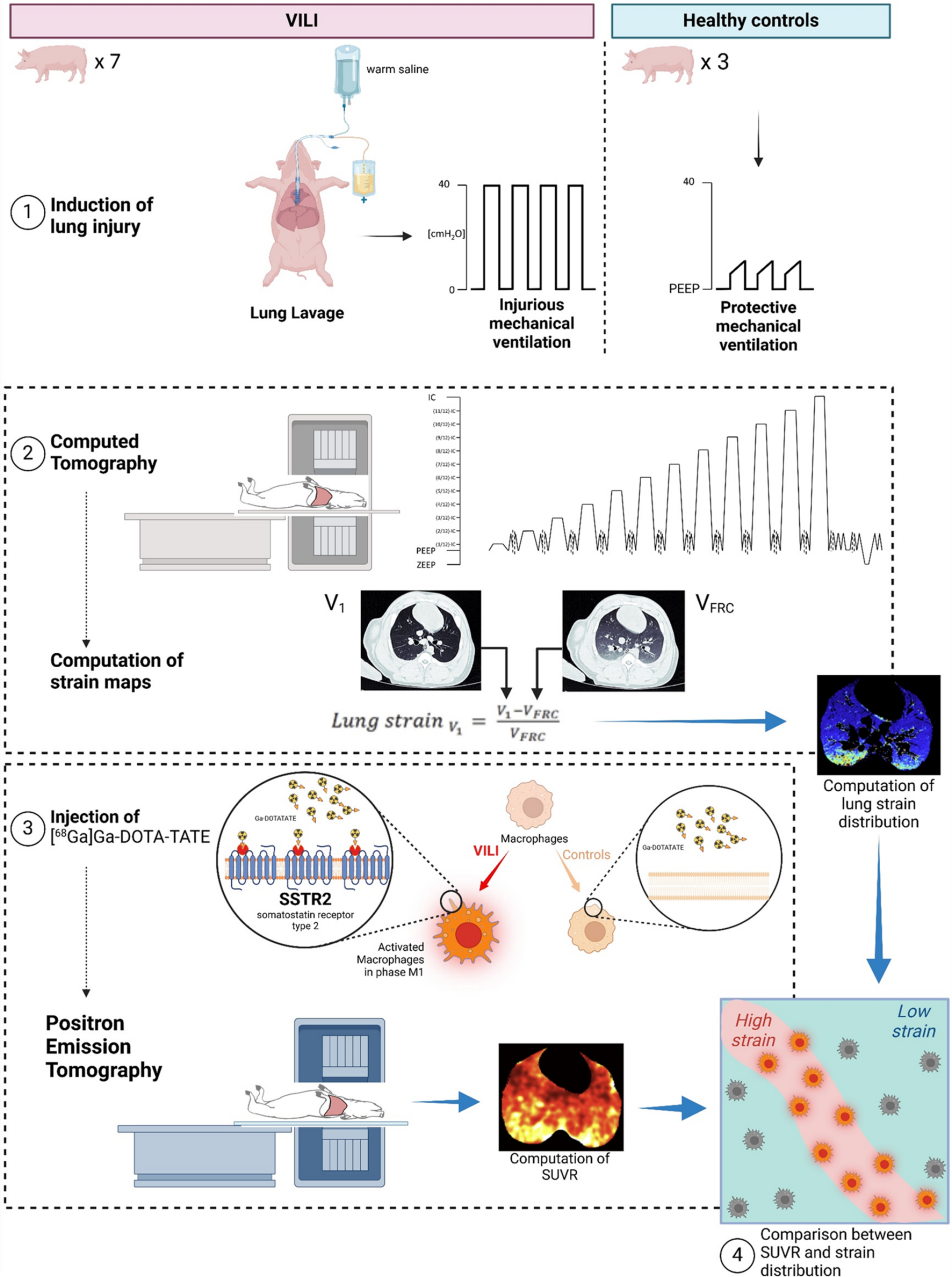
Outline of the experiment. Phase 1. Two groups of animals were studied: one underwent a dual-hit lung injury composed of lung lavage followed by injurious mechanical ventilation; the other group consisted of healthy controls. Phase 2. All the animals underwent the computed tomography of the entire lung at different lung volumes between ZEEP and inspiratory capacity (IC). The map of lung strain distribution was obtained by subtracting voxel-by-voxel the gas content at ZEEP (here labeled as *V*_FRC_, volume at functional residual capacity—FRC, assuming that the end-expiratory lung volume at ZEEP can be considered practically equivalent to FRC in static conditions and in absence of intrinsic PEEP) from the gas content at IC. The difference was divided by *V*_FRC_: more details in the text. In phase 3, with the animal still in the PET–CT scanner, we administered the tracer [^68^Ga]Ga-DOTA-TATE, which has a high affinity for the SSTR2 receptors expressed on the surface of activated macrophages during inflammatory processes. The standardized uptake value ratios of the mentioned tracer allowed us to assess the regional distribution of activated macrophages. In phase 4, using information theory, we compared whether the activated macrophages foregather in the areas where the distribution of strain is higher (picture created with BioRender.com)

The animal experiments were authorized by the Animal Research Ethics Committee (decision number 5.8.18-15648/2019) of Uppsala Region and carried out according to institutional guidelines (“Uppsala university guidelines on animal experimentation”, UFV 2007/724) and reported according to the ARRIVE guidelines [20]. The timeline of the experiment is presented in Fig. 1 and the outline of the experimental protocol is illustrated in Fig. 2.

### Anesthesia induction and animal preparation

Piglets (*n* = 10; 7 cases, 3 controls; weight 24.6 ± 1.6 kg) were pre-medicated through intramuscular injection of tiletamine 2.2 mg/kg and Zolazepam 6 mg/kg and lifted on the operatory table once unconscious. Subsequently, a peripheral intravenous line was established while peripheral blood saturation and 3 derivation ECG monitoring was applied. Fentanyl (1.5–2 mcg/kg) was administered intravenously before performing surgical tracheostomy and airway was secured with endotracheal cuffed tube (ETT) number 6 (6.0 Hi-Contour; Mallinckrodt Medical, Athlone, Ireland).

General anesthesia was maintained with intravenous Ketamine 32 mg/kg/h, Fentanyl 4 μg/kg/h, and Midazolam 0.12 mg/kg/h in Glucose solution 25 mg/mL. Muscle relaxation was achieved with intravenous Rocuronium 2.5 mg/kg/h. Mechanical ventilation was started with a tidal volume (*V*_T_) 8 ml/kg, respiratory rate (RR) 30 breath/min; positive end-expiratory pressure (PEEP) 5 cmH_2_O, F_I_O_2_ 0.5. A high-flow central venous catheter, an arterial catheter, a pulmonary artery catheter, and a suprapubic urinary catheter were positioned via open surgical techniques. An esophageal catheter was positioned in the distal third of the esophagus to obtain a continuous measurement of esophageal pressure (*P*_es_). Another balloon catheter was positioned in the gastric lumen, and continuous measurement of gastric pressure (*P*_ga_) was obtained. The correct position of the esophageal and gastric catheters was ascertained using a C-arm portable radiological machine. Hemodynamic advanced monitoring consisted of invasive arterial pressure, central venous pressure, pulmonary arterial pressure, wedge pressure, and cardiac output (this last by the technique of the cold bolus of saline executed in triplicate, injected randomly during the respiratory cycle). The animals were then ventilated for 30 min without performing any procedure to allow for stabilization.

### Instrumental setup for lung mechanics measurements

Airway flow was measured continuously with a Fleisch pneumotachograph (Laminar Flow Element type PT, Special Instruments GmbH, Nördlingen, Germany) installed between the ETT and the ventilator, and connected to a differential pressure transducer (Diff-Cap Pressure Transducer, Special Instruments GmbH, Nördlingen, Germany). Pressure at airway opening, *P*_es_, and *P*_ga_ were measured continuously via three pressure transducers (Digimaclic Pressure Transducers, Special Instruments GmbH, Nördlingen, Germany). All respiratory signals were acquired via a data acquisition system (PowerLab PL 16/35, ADInstruments, Dunedin, New Zealand) controlled by the LabChart Software (ver. 7.0, ADInstruments, Dunedin, New Zealand). The inspiratory and expiratory airway volumes were obtained via flow integration.

### Lung injury

Acute lung injury was induced in 7 of 10 animals using a “two-hit” procedure, combining lung lavages with warm saline (30 ml/kg sodium chloride 9 mg/ml at 37 °C), followed by injurious mechanical ventilation with high tidal volume and low PEEP [10]. Lung lavages were repeated to reach a PaO_2_/F_I_O_2_ ratio (*P*/*F*) ≤ 200 mmHg. Subsequently, injurious mechanical ventilation was started and maintained for a minimum of 30 min and a maximum of 60 min until a *P*/*F* ratio < 150 mmHg was reached. It consisted of pressure controlled mechanical ventilation with applied airway pressure 40 cmH_2_O; ZEEP (zero end-expiratory pressure); RR 30 breath/min; F_I_O_2_ = 1.

After the induction of VILI, protective ventilation was initiated (baseline ventilation) with a *V*_T_ of 6 ml/kg. PEEP was adjusted according to the ARDSnet F_I_O_2_/PEEP table [21], aiming to a SpO_2_ target of 89–95%. The respiratory rate was set aiming to normocapnia. This was the ventilation mode throughout the entire experiment, with the temporary exception relative to the CT exposure at different inflation volumes. Animals belonging to the control group underwent directly protective ventilation without lung injury. The total time of mechanical ventilation was equal for both the control and VILI group of animals.

### CT-imaging

To estimate the regional distribution of lung strain at different inflation volumes, we used an already-established procedure [7] that we summarize here following.

The first step was to measure spirometrically the inspired volume by applying a constant pressure of 40 cmH_2_O for 20 s, which was denoted as inspiratory capacity (IC). Based on this, CT scans of the lungs were performed at 12 different inflation volumes, corresponding to incremental steps of 1/12 of the IC. Subsequently, this value was divided into twelve volumetric steps (*V*_T_ = (IC/12) × 1, (IC/12) × 2, (IC/12) × 3… up to IC) and noted down.

The animals were moved to the PET Center on a specially designed trolley, ventilated by the same ventilator and the baseline ventilation described before. After positioning on the PET–CT scanner bed in a supine position, they underwent RM at 40 cmH_2_O for 40 s to eventually restore the history of volumes. The twelve incremental *V*_T_ steps were then delivered sequentially, each followed by an inspiratory hold maneuver (IHM) of approximately 10 s, during which an attenuation CT scan of the entire lung was acquired. Each IHM was followed by 2–3 min of tidal breathing to allow for the restoration of mechanical steady-state conditions. After IC was reached, two more CT scans were acquired during end-expiratory hold maneuvers (EHMs) at PEEP and ZEEP conditions (Fig. 2). The 64-slice CT scanner used a digital 4-ring system and had a 198-mm axial field of view (FOV) and the following parameters: 100 kV, 80–400 mA, noise index 10, rotation 0.5″, full spiral, slice thickness 3.75 mm, pitch 0.98:1, recon diameter 50 mm.

### Measurements of hemodynamics

A further coordinated set of measurements was performed three times during the experiment: at baseline after animal preparation (T1), after VILI induction (T2), and just before the piglet was euthanized (T3). These measurements consisted of arterial blood gas (ABG) analysis and the above-mentioned hemodynamic measurements. At the end of the protocol, the animals were euthanized under deep anesthesia with an intravenous bolus of potassium chloride.

### Lung strain regional distribution maps

For each animal, two transverse planes per inflation volume were analyzed. One corresponded to a para-diaphragmatic plane, located 2 cm cranially to the diaphragm; and one mid thoracic transverse plane, equidistant from lung apex and diaphragmatic dome.

Utilizing purposely written MatLab scripts by the authors (GP, MP), the perimeter of the lung parenchyma was manually contoured for each analyzed CT slice. This was done to avoid potential errors that automatic segmentation software may introduce, especially when analyzing consolidated lung areas [22]. To preserve the correspondence between voxels deriving from images shot at different inflation volumes, a lung image registration [23] procedure was applied before comparing images. It was based on purposely written scripts for the Image Processing Toolbox of MatLab (MatLab R2022b, The MathWorks, Natick, USA) and already validated in previous research by our group [22, 24].

For each image, a corresponding volume map was created by transforming the HU densities into gas volume at a voxel level, according to the concept proposed by Gattinoni et al. in 1988 [25]:

1 $$V_{{{\text{vox}}, {\text{gas}}}} = V_{{{\text{vox}}}} \cdot \frac{{ - {\text{HU}}}}{1000},$$

where *V*_vox_ is the volume of a voxel, *V*_vox_,_gas_ is the volume of gas contained in the voxel, HU is the measure of the density in Hounsfield units. This method operates by approximating the density of a fully aerated area of lung parenchyma to air and the density of a fully consolidated area of lung parenchyma to water. Following this assumption, each voxel is assigned a gas content percentage that is directly proportional to its HU number in a spectrum between the values of HU for gas and water.

The Hookean strain is a measure of displacement of an elastic body from its equilibrium point: if the lung volume at Functional Residual Capacity (*V*_FRC_) is considered the elastic equilibrium point of the lung, the proportion of strain acting on the lung, at a given inflation volume (*V*_1_) can be written as [26]

2 $${\text{Lung}}\;{\text{strain }}_{{V_{1} }} = \frac{{V_{1} - V_{{{\text{FRC}}}} }}{{V_{{{\text{FRC}}}} }}.$$

We applied Eq. 2 to all the voxels corresponding to lung parenchyma. By hypothesizing that strain is (a) homogenously distributed inside each voxel and (b) all deformation corresponds to *normal* strain (i.e., perpendicular to the faces of the voxels), it is possible to quantify the voxel-associated Normal Strain (VoStrain) [27]:

3 $${\text{VoStrain}}_{{i, V_{1} }} = \frac{1}{3} \frac{{V_{i,1} - V_{{i,{\text{FRC}}}} }}{{V_{{i,{\text{FRC}}}} }}.$$

This allowed us to represent the VoStrain associated with the different parts of the lung (*field of strain, FOS*) as computed from the CT scans.

For voxels belonging to completely atelectatic areas of the lung at FRC (i.e. presenting a null content of gas) an arbitrary gas content of 0.01% of voxel volume was assigned, to avoid values of VoStrain → ∞ in the computation of the field of strain. The strain that was analyzed during the successive part of the study was the one that the animals of the VILI group faced during the injurious mechanical ventilation, i.e., the strain developed ventilating between ZEEP and 40 cmH_2_O, while for the healthy controls, it was the strain corresponding to their end-inspiratory volume.

#### PET imaging

Immediately after having completed the acquisitions of CT images, the pigs were injected intravenously with a target dose of 2 MBq/kg of [^68^Ga]Ga-DOTA-TATE and underwent, in the same scanner (Discovery MI, GE Healthcare) a dynamic PET scan of 60 min (30 frames: 12 × 10 s″, 6 × 30 s″, 5 × 2 min′, 5 × 5 min′, 2 × 10 min′) while mechanically ventilated with the same pattern of breathing described before. The PET and CT acquisitions were automatically co-registered, as the pigs were not moved between the scans.

The PET images were generated by computing the standardized uptake value ratio (SUVR) [28] of the lung tissue, computing the ratio of radioactivity concentration (expressed as MBq/mL) between each lung voxel (*c*_voxel_) at the coordinates *x*, *y* and the average concentration inside the left ventricle of the heart (*c*_ref_) within the same PET image, at 3596 s (≈ 1 h) from the injection of [^68^Ga]Ga-DOTA-TATE:

$${\text{SUVR}}_{x,y} = \frac{{c_{{{\text{voxel}}_{x,y} }} }}{{c_{{{\text{ref}}}} }}.$$

This allowed us to draw the regional distribution of SUVR at voxel level. The images depicting the SUVR distribution corresponding to the two chosen transversal planes of the CT-derived strain maps were further analyzed.

### Image analysis and statistics

The field of strain and the SUVR of corresponding lung sections were further assessed. The images were analyzed using descriptive statistics (as mean ± standard deviation and median with interquartile range of all the animals and separately for VILI and control animals) and inferential statistics of FOS and SUVR. We could not exclude a non-normal distribution of the studied variables: for this reason, we used the nonparametric Wilcoxon signed rank test (*α* = 0.05) throughout the present manuscript, except where explicitly stated. The following question were addressed:

*Q1. Whether the SUVR of the PET images pertaining to controls and VILI have the same median;*

*Q2. Whether the FOS derived from CT images pertaining to controls and VILI have the same median;*

After having divided all the PET and the FOS images into 100 consecutive iso-gravitational layers and having computed the means at each gravitational level from the four groups of animals (four conditions: mid-thorax or para-diaphragmatic; VILI or healthy controls) we tested whether:

*Q3. The gravitational course of the SUVR from PET images pertaining to controls and VILI are equal*

*Q4. The gravitational course of the FOS images pertaining to controls and VILI are equal*

by performing the linear regression of the progressively leaning part of the curves (corresponding to the two central quartiles of the distribution, i.e. from slice 25 to 75) and evaluating whether the regression lines subtended different models by applying the *F* test [29].

To ascertain:

*Q5. Whether the local amount of activated macrophages is determined only by the distribution of blood due to gravitational forces,*

We measured the relation between the volume of nongaseous component per each voxel (corresponding to the volume of blood or tissue) and SUVR, separately for diaphragm and for mid-thorax level, in healthy and VILI. This calculation of nongaseous content was performed using the method described by Pelosi et al. [30]. We computed the linear regressions between SUVR and nongaseous content deriving from the leaning portion of the two distribution profiles in the four conditions mentioned above. We compared the angular coefficients during VILI with the corresponding healthy conditions by computing the *Z*-statistic, according to Paternoster et al. [31].

Then, we compared the informative content between each pair of corresponding images, obtained with a different modality (e.g. FOS vs. SUVR pertaining to the same level, the same animal and separately for controls and *VILI* animals). For each couple of images, we computed the Linear Mutual Information (LMI) according to the method described by Lange and Grubmüller [32], in order

*Q6. To verify that the information contained in one of the modalities was also present in the other*.

By testing whether LMI was statistically different between the mentioned groups (Wilcoxon rank sum test, *α* = 0.05). The above-described image analysis and statistical testing were conducted using scripts specially developed by the authors GP and SS, for the Image Processing and the Statistics and Machine Learning Toolbox of MatLab (MatLab R2022b, The MathWorks, Natick, USA).

## Results

All the animals survived the experiment. They presented at baseline a *P*/*F* ratio of 531.0 ± 43.4 mmHg. The pigs that underwent lung injury reached a *P*/*F* ratio of 79.0 ± 30.8 mmHg (*p* = *0.01*) while the controls remained stable at *P*/*F* ratio 464.0 ± 44.5 mmHg (*p* = *0.25*). The main cardiorespiratory data are presented in Table 1. The animals belonging to the VILI group were ventilated (during the phase of injurious ventilation) with a tidal volume of 436.6 ± 134 [ml], which corresponded to 18.0 ± 5 [ml/kg].

**Table 1:** Cardiorespiratory variables during the three main phases of the experiment in the two groups of healthy controls and animals exposed to ventilator-induced lung injury (VILI)

Time	T1		T2		T3	
	Healthy	VILI	Healthy	VILI	Healthy	VILI
Heart rate [bpm]	94.3 ± 13.1	98.9 ± 14.7	84.3 ± 5.3	88.6 ± 14.7	86.0 ± 3.7	95.0 ± 13.1
Systemic systolic pressure [mmHg]	102.3 ± 13.6	88.7 ± 7.8	104.7 ± 10.9	86.1 ± 5.2*	103.0 ± 4.2	103.4 ± 11.2
Systemic diastolic pressure [mmHg]	71.3 ± 11.6	57.3 ± 6.1	71.0 ± 7.8	56.0 ± 6.3*	63.3 ± 1.2	65.0 ± 7.3
Pulmonary systolic pressure [mmHg]	22.7 ± 2.4	22.9 ± 3.8	28.0 ± 0.0	31.6 ± 10.3	28.7 ± 3.1	36.6 ± 8.0
Pulmonary diastolic pressure [mmHg]	10.7 ± 3.7	10.7 ± 2.2	11.3 ± 0.9	17.6 ± 7.1	11.7 ± 3.4	24.1 ± 10.5
Wedge pressure [mmHg]	9.0 ± 1.4	7.7 ± 1.8	9.3 ± 1.9	8.7 ± 2.3	9.7 ± 1.2	12.0 ± 2.4
Central venous pressure [mmHg]	8.3 ± 1.2	6.0 ± 1.8	8.0 ± 1.4	7.7 ± 2.2	9.7 ± 2.9	9.7 ± 2.8
Cardiac output [l/min]	2.9 ± 0.9	2.5 ± 0.5	2.7 ± 0.3	2.6 ± 0.6	3.1 ± 0.5	3.3 ± 0.4
Tidal volume [ml]	243.3 ± 20.5	227.1 ± 8.8	190.0 ± 8.2	202.9 ± 16.0	203.3 ± 4.7	221.7 ± 22.7
Respiratory rate [bpm]	31.7 ± 2.4	31.4 ± 3.5	35.0 ± 0.0	35.7 ± 3.2	35.0 ± 0.0	36.7 ± 2.4
PEEP [cmH_2_O]	5.0 ± 0.0	5.0 ± 0.0	5.0 ± 0.0	8.7 ± 2.1*	5.0 ± 0.0	9.8 ± 1.5*
Minute volume [l/min]	7.6 ± 0.8	7.5 ± 1.0	6.9 ± 0.2	7.4 ± 1.2	7.5 ± 0.3	8.3 ± 0.9
Mechanical power [J/min]	11.1 ± 2.3	11.1 ± 2.2	9.8 ± 0.2	17.1 ± 5.0	10.5 ± 0.4	21.4 ± 4.0*

The three times of data sampling were: at baseline after animal preparation (T1), after VILI induction (T2), and just before the piglet was euthanized (T3). The asterisk marks a statistically significant difference (Student *t* test, *α* = 0.05) between healthy controls and VILI

Their total lung compliance went from 21.6 ± 4.9 to 12.2 ± 2.6 [ml/cmH_2_O] in the animals subjected to lung injury (*p* = *0.01*) and from 25.6 ± 6.5 to 25.3 ± 6.0 (*p* = *0.75*) for the controls.

The analysis of respiratory mechanics showed that when all the animals were examined at the same reference volume of (IC/12) × 6, corresponding to 273.6 ± 87.8 [ml], the healthy controls showed a *P*_es_ 14.7 ± 5.0 [cmH_2_O], a transpulmonary pressure (*P*_TP_) of 4.0 ± 5.9 [cmH_2_O] and a driving pressure (Δ*P*) of 13.7 ± 1.6 [cmH_2_O], while the animals subjected to VILI presented *P*_es_ 13.1 ± 2.7 [cmH_2_O], *P*_TP_ of 12.9 ± 4.3 [cmH_2_O] and Δ*P* of 18.0 ± 4.0 [cmH_2_O]. Statistical analysis comparing healthy controls with VILI group reported a significant difference in terms of *P*_TP_ (*p* = *0.03*), while the difference in *P*_es_ and Δ*P* did not reach statistical significance (respectively *p* = *0.58* and *p* = *0.13*).

**Fig. 3:**
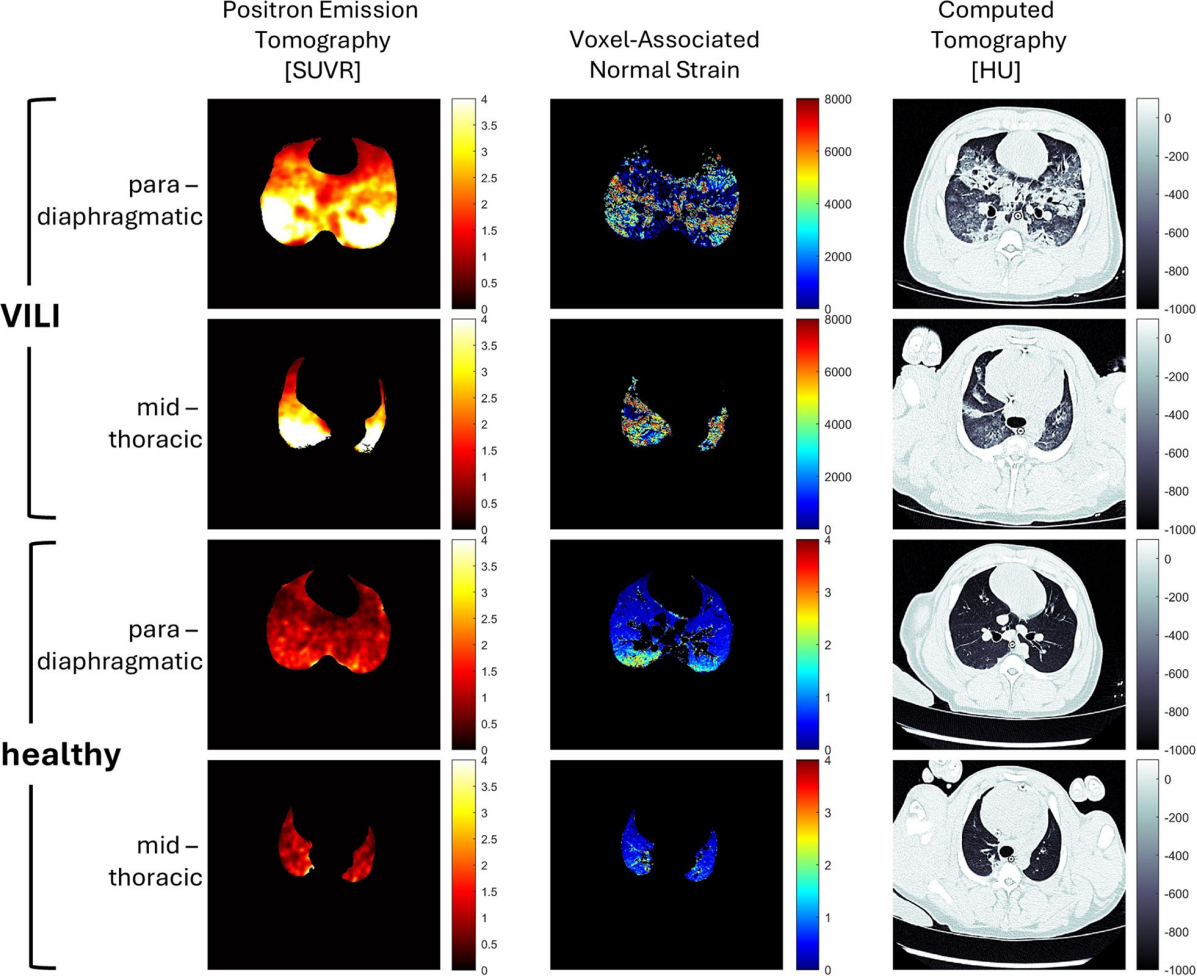
Representative picture of standardized uptake value ratio (SUVR), voxel-associated normal strain (VoStrain) distribution and computed tomography (CT) in one animal of the VILI group and one healthy control, at para-diaphragmatic and mid-thoracic levels. SUVR and strain are dimensionless measures. HU: hounsfield number. Please note that the color scale of the VoStrain is different between VILI and healthy controls: the regional voxel-associated normal strain in VILI can reach levels three orders of magnitude higher than in healthy controls

The computation of strain was performed between lung volume at ZEEP and the highest inflation volume to which the animals were exposed during the induction of VILI, corresponding to the inspiratory capacity. By this way the experiment produced 21,300 CT scans, we selected 240 CT slices and brought to the final analysis 40 images (10 animals × 2 slice levels × 2 inflation volumes), see Fig. 3.

### Strain

We computed the VoStrain to which the animal was exposed during the induction of VILI, using the CT images obtained at inspiratory capacity and the CT images taken at ZEEP. This was calculated at para-diaphragmatic level and at mid-thoracic level. Data are reported as mean ± standard deviation, followed by (median; interquartile range).

For the VILI group, average VoStrain over the entire slice was 315 ± 79 (284; 181) at para-diaphragmatic level and 185 ± 165 (122; 155) at mid-thoracic level. For the control group, it was respectively: 108 ± 6 (104; 52) and 24 ± 4 (21; 22).

**Fig. 4:**
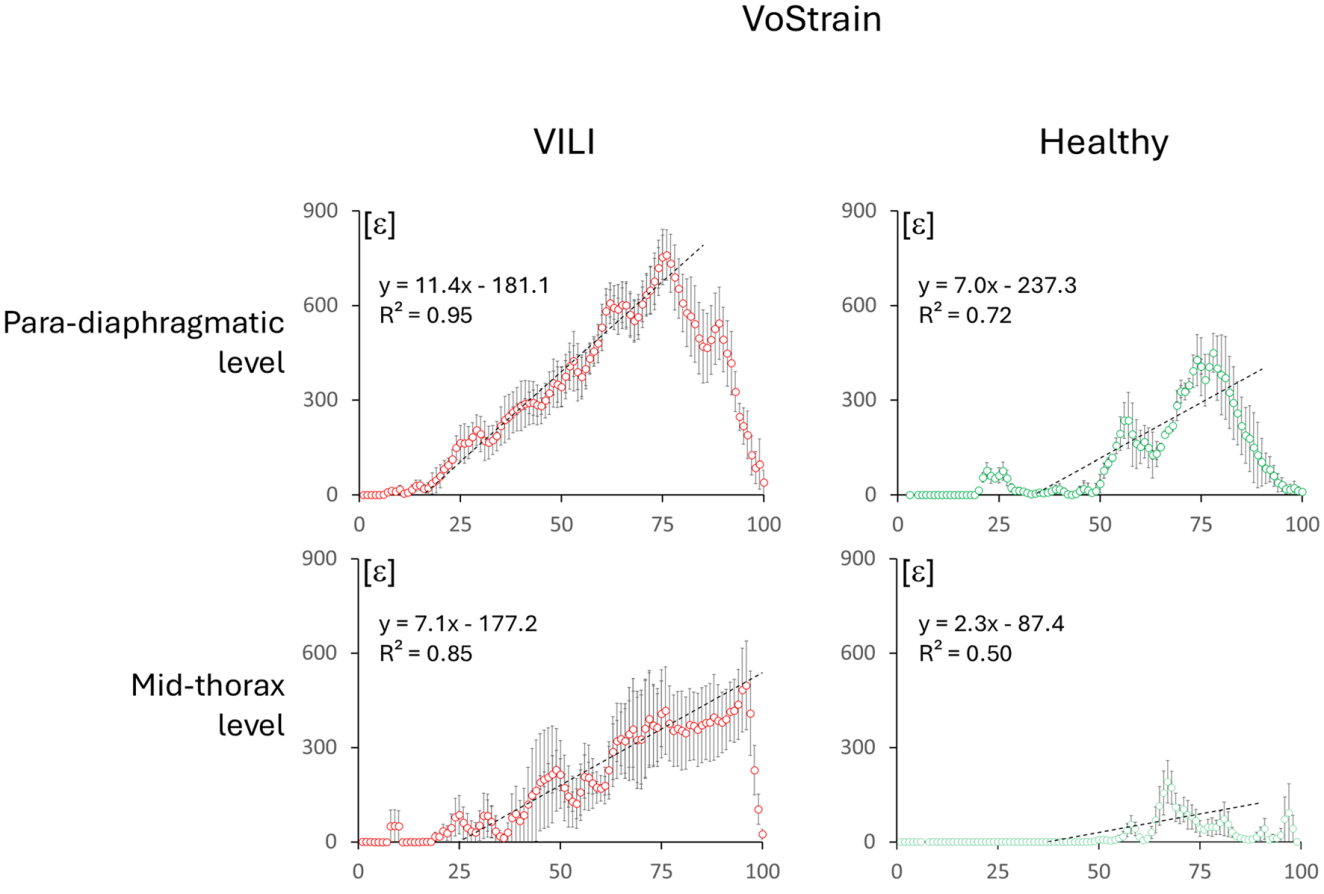
VoStrain in the different groups of analyzed images: in VILI and healthy controls, at para-diaphragmatic and mid-thoracic levels. The graphs represent the mean ± standard error obtained by averaging the 100 iso-gravitational slices of the lung across the different animals. The dashed line depicts the linear regression of the leaning part of the profiles, for homogeneity identified as the central quartiles (from slice 25 to 75) in all the graphs. The graph also reports the respective regression lines and regression coefficients. *ε* represents VoStrain and it is a dimensionless variable

Plotting the profile of VoStrain along the gravitational axis (*x*-axis in Fig. 4) it is possible to observe that it reaches a maximum of 760 ± 210 (812; 268) at para-diaphragmatic level, while at mid-thoracic level max value was 497 ± 373 (668; 582); in the controls it was respectively: 449 ± 106 (485; 131) and 193 ± 160 (255; 101).

The gravitational course of strain, analyzed using regression lines, showed significant differences between healthy and VILI conditions at both mid-thorax (*p* < 0.01) and para-diaphragmatic levels (*p* < 0.01).

### PET

The animals received an average dose of 2.3 ± 0.9 MBq/kg of [^68^Ga]Ga-DOTA-TATE. After the PET study, we gathered a total of 2130 images (10 animals × 30 times × 71 sections). The following data are reported as mean ± standard deviation, followed by (median; interquartile range). The mean SUVR for entire images at para-diaphragmatic level in healthy conditions was 0.6 ± 0.0 (0.6; 0.0); after lung injury it was 1.1 ± 0.6 (0.9; 0.7); at thorax level, it was, respectively, 0.3 ± 0.0 (0.3; 0.1) in healthy and 0.5 ± 0.3 (0.3; 0.4) after injury.

**Fig. 5:**
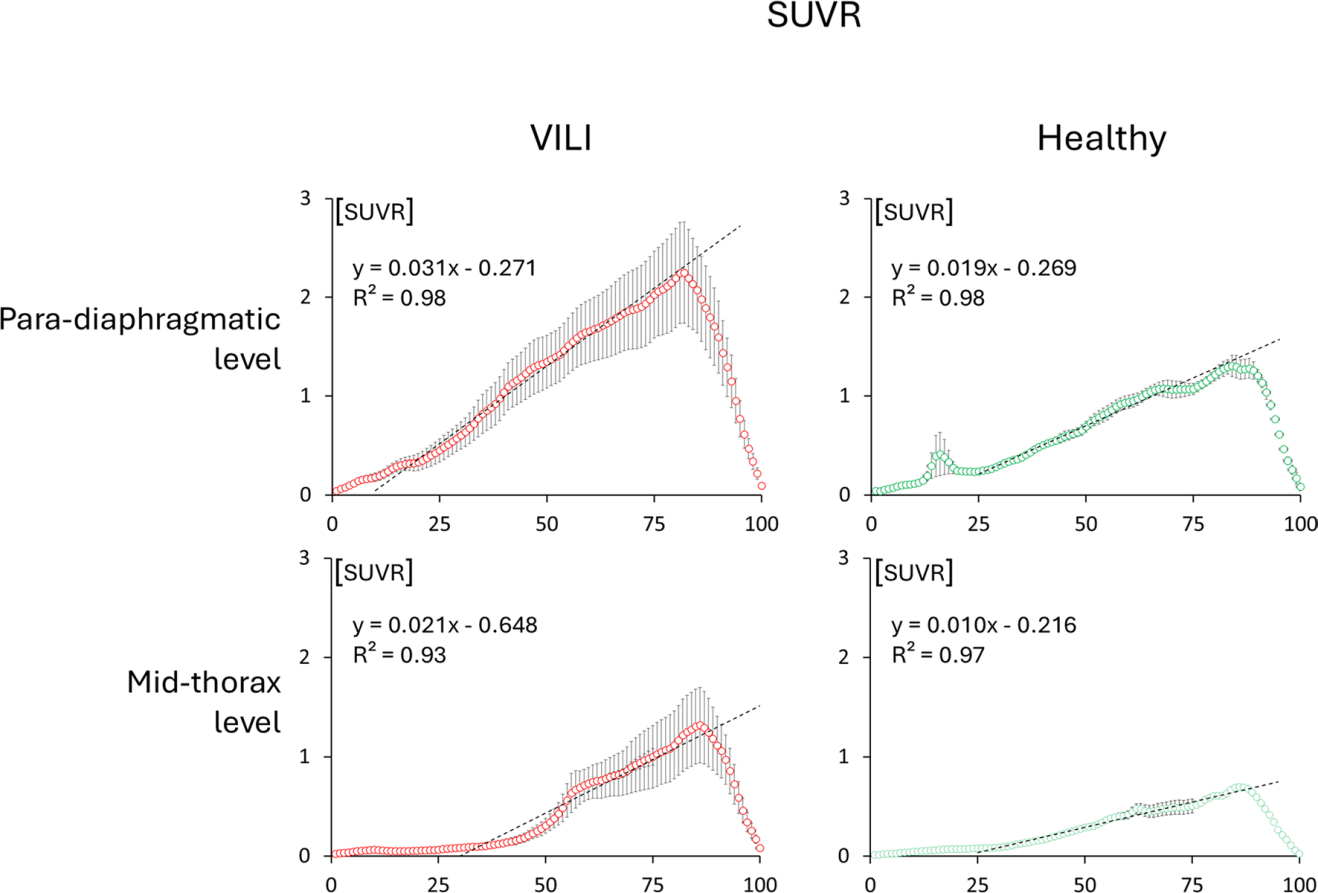
Standardized uptake value ratios (SUVR) in the different groups of analyzed images: in VILI and healthy controls, at para-diaphragmatic and mid-thoracic levels. The graphs represent the mean ± standard error obtained by averaging the 100 iso-gravitational slices of the lung across the different animals. The dashed line depicts the linear regression of the leaning part of the profiles, for homogeneity identified as the central quartiles (from slice 25 to 75) in all the graphs. The graph also reports the respective regression lines and regression coefficients. SUVR is a dimensionless variable

The topographic distribution of SUVR (see Fig. 5) of [^68^GA]GA-DOTA-TATE was higher in the dependent regions and reached a maximum of 1.3 ± 0.1 (1.3; 0.1) at para-diaphragmatic level in controls; at the same level, it was 2.2 ± 1.3 (1.8; 1.6) during VILI; at mid-thorax it was 0.6 ± 0.03 (0.7; 0.0) in controls and 1.3 ± 1.0 (0.8; 1.3) in VILI.

The profiles of the regression lines describing the increase of SUVR along the gravitational axis showed that at both mid-thoracic and para-diaphragmatic levels, the curves followed statistically different regression lines between VILI and healthy conditions at both mid-thoracic and para-diaphragmatic level, both expressed by *p* < 0.01.

### SUVR vs. nongaseous content of the images

**Fig. 6:**
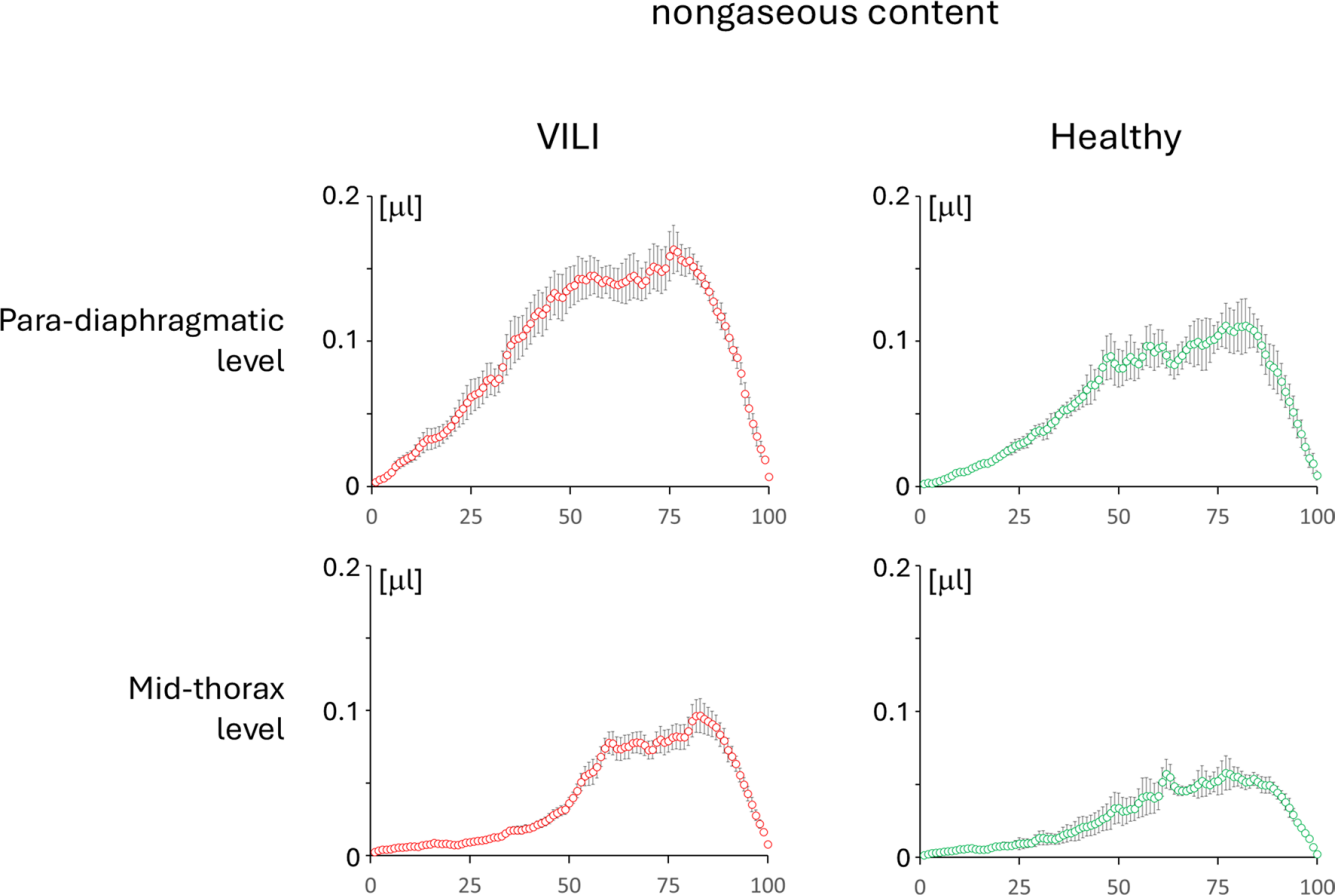
Nongaseous content in the different groups of analyzed images: in VILI and healthy controls, at para-diaphragmatic and mid-thoracic levels. The graphs represent the mean ± standard error obtained by averaging the 100 iso-gravitational slices of the lung across the different animals. Nongaseous content is represented in microliters [µl]

**Fig. 7:**
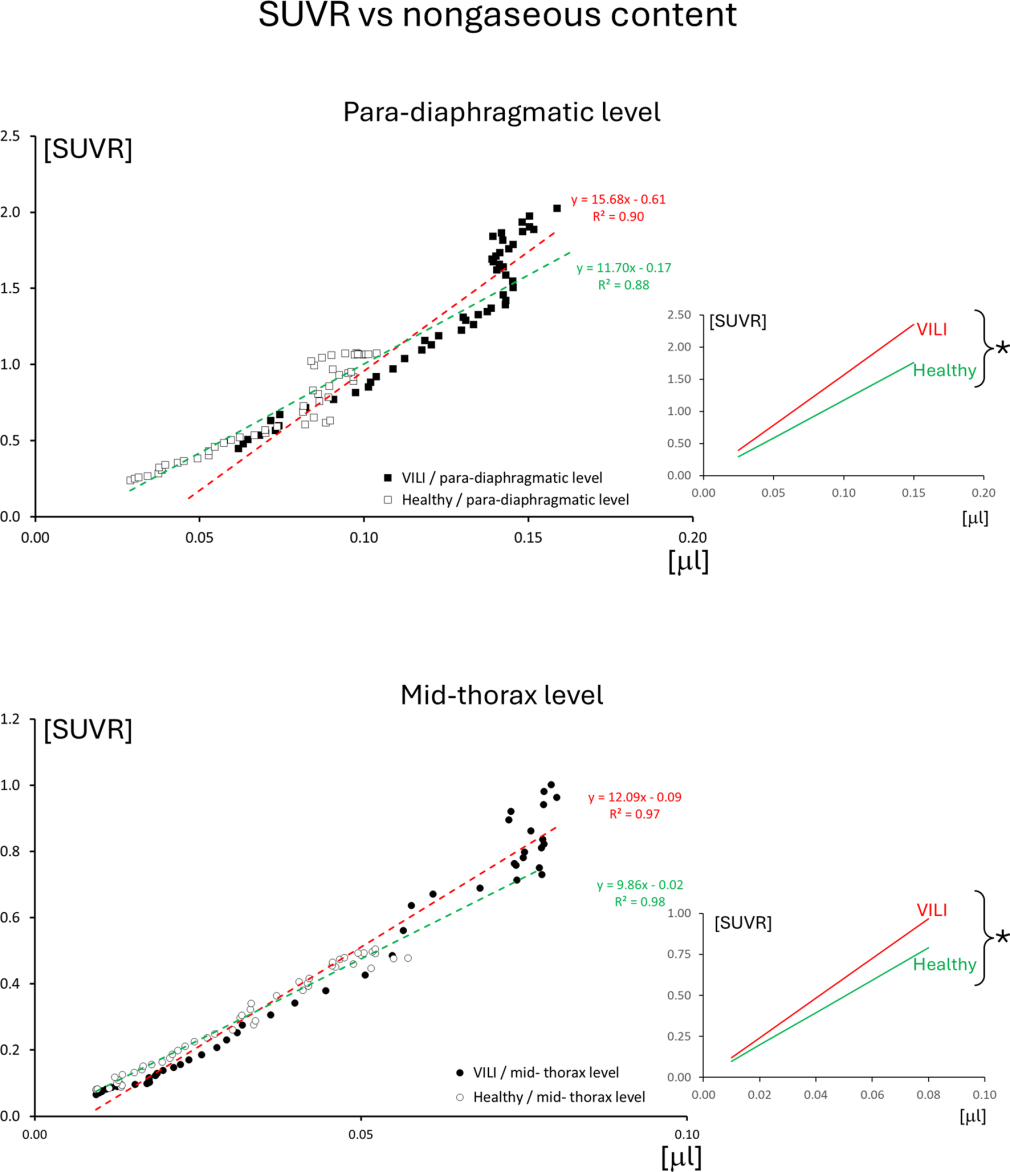
Scatter plot of SUVR vs. nongaseous content, deriving from the same iso-gravitational slices, sampled in the leaning part of both distribution profiles. The two panels compare VILI vs. healthy controls at para-diaphragmatic and mid-thorax levels. The respective linear regression equations are also represented (regression lines for VILI are represented in red and for healthy in green). The rate of increase of SUVR and non-gaseous content along the gravitational axis is represented by the angular coefficients of the regressions. For clarity, on the right of the scatter plots, it is reported the relation between the same regression lines, imposing an intercept of zero, to facilitate the comparison of the angular coefficients. The *Z* tests performed on both levels (VILI vs. healthy at para-diaphragmatic and mid-thoracic levels) yielded a statistically significant difference between angular coefficients (marked with “*”), demonstrating that the rate of increase of SUVR is higher in VILI than in healthy. This means that at the same amount of nongaseous content in the lung (that in the VILI group may derive from excess tissue as in atelectasis or from a higher local perfusion due to vasodilation and inflammation), the concentration of macrophages that are present on-site is higher than the one that can be considered “passively carried” by blood flow

The course of the nongaseous content is presented in Fig. 6 and, as expected, increases progressively in the most dependent regions of the lung. The regression lines (see Fig. 7) of SUVR (*y*) vs. amount of nongaseous component (*x*) yielded for para-diaphragmatic level in VILI: *y* = 15.68*x* − 0.61 (*R*^2^ = 0.90); for para-diaphragmatic level in healthy: *y* = 11.70*x* − 0.17 (*R*^2^ = 0.88); for mid-thorax in VILI: *y* = 12.09*x* − 0.09 (*R*^2^ = 0.97); for mid-thorax in healthy: *y* = 9.86*x* − 0.02 (*R*^2^ = 0.98). All the regression equations were statistically significant as well as there was a statistically significant difference (by applying the *Z* test on the angular coefficients) between healthy and VILI at both diaphragm level (*p* = 1.6 × 10^–5^) and mid-thorax level (*p* = 6.8 × 10^–9^).

### Mutual information

Computation of the linear mutual information between FOS and SUVR images yielded a global 0.734 ± 0.144 during VILI and for controls 0.420 ± 0.162. Their difference was statistically significant with *p* = 6·10^–4^. In particular, at the para-diaphragmatic level, it presented 0.793 ± 0.108 in VILI and 0.564 ± 0.046 in healthy conditions (statistically different, *p* = 0.017); at the mid-thorax level, it was: during VILI 0.676 ± 0.159 and in healthy conditions: 0.275 ± 0.017 (statistically different, *p* = 0.017).

## Discussion

This study demonstrated that in an animal model of VILI induced by surfactant depletion and injurious ventilation, activated macrophages plausibly in phase M1 accumulate in the areas that were subjected to high local strain. These areas can be located according to the classical ventilation distribution of the lung or in proximity of closed/atelectatic parenchyma because exposed to high traction force [33]. This is the first time this relation has been examined on a voxel-by-voxel basis in a multi-modal image analysis.

First, we have separately analyzed the distribution of strain and SUVR for [^68^Ga]Ga-DOTA-TATE. We have observed that for both the techniques, the images pertaining to healthy controls and VILI had different medians, presenting the images in VILI a higher strain and a higher SUVR than healthy lungs (hypothesis Q1 and Q2).

Then, we studied separately the distribution of both strain and SUVR along the gravitational axis, by dividing the images in 100 isogravitational levels. With both methods (SUVR and FOS) there was a statistically significant difference between the regression lines subtending the behavior in healthy and VILI conditions (hypothesis Q3 and Q4).

The meaning of these findings resides in the fact that the strain and the SUVR increase in the most dependent areas of the lung, growing differently in healthy and VILI conditions. The gravitational increase of strain confirms the well-established concept that ventilation is higher in the most dependent areas of the lung [34], as well as the hypo- or non-ventilated areas, so consequently, there is the potential for a higher strain [7]. Moreover, the difference between the inclinations of the two regression lines of SUVR in healthy and VILI conditions (Fig. 7), suggests that the higher presence of macrophages in the dependent areas is not a simple consequence of gravitational forces. To confirm this, we have measured the regression lines between SUVR and the amount of nongaseous component of each voxel (composed of fluids, like blood and of tissues, like atelectatic lung). If the distribution of macrophages (mirrored by the SUVR) followed only a gravitational distribution, the ratio between SUVR and nongaseous component should have followed the same equation line, independently if the overall amount of SUVR was high or low. However, the comparison has demonstrated that the equations subtending SUVR vs. nongaseous component are different between healthy and VILI condition, supporting the idea that the accumulation of macrophages is not a simple consequence of gravitational forces.

We hypothesize that the SUVR of [^68^Ga]Ga-DOTA-TATE portrays activated macrophages polarizing toward the M1 phenotype and reflects an ongoing inflammatory process. It is still matter of debate the proportion between resident (intra-alveolar and interstitial) macrophages and the latterly arrived on the site of inflammation by the recruitment of circulating monocytes [35]. A classical view is that the overall amount of blood is higher in the gravitational dependent areas [36], however, is equally clear that the regional distribution of blood depends also on local factors [37]. This means that the arrival of recruited monocytes–macrophages can follow also other pathways than the gravity-paved one.

Strain derived from CT images is a measure of the displacement caused by the force field, and PET depicts the distribution of a tracer inside the lung.

In a second phase of our study, to make a voxelwise comparison between the distribution of strain and of activated macrophages, we applied Shannon’s mutual information laws, included in Lange and Grubmüller method [32]. To compare these couples of images (strain map derived by CT and macrophage map obtained by SUVR and [^68^Ga]Ga-DOTA-TATE) it is necessary not only to see whether there is a generic association between them but also to measure whether they express the same informative content.

Many methods are available [38] to perform this task. Shannon’s mutual information is one of the most efficient and most widely used methods for calculating the similarity between images obtained with different modalities. Based on Shannon’s theorem of information [39] and qualitatively described, it yields the probability that the intensity of corresponding pixels (in two images obtained with a different technique) shows a coordinated variation, thus depicting the same objects. Measurement of mutual information in our images demonstrated that activated macrophages converge in high numbers where the strain is high (Q6). In fact, during VILI, PET images detect many more activated macrophages that gather in the same position where there is high strain. For this reason, these “flashes of light” that PET images show during VILI *inform* the viewer that a higher intensity is present in the corresponding strain maps. This information is mutual: analyzing strain map intensity reveals the location of activated macrophages.

### Computation of voxel-associated strain

Differently from the “spirometrical” strain computed at airways opening [40], the voxel-associated strain (VoStrain) is computed from a change of density determined by a CT [41, 42]

Assuming that each voxel subtends anatomical structures that have the same behavior, the voxel-associated strain yields the amount of strain that is distributed on each of the three axes of the voxel and is defined by classical physics of elastic materials [27]. Assigning an amount of strain to each axis makes it possible to deduce the magnitude of deformation to which molecules composing the so-called stress-bearing elements are exposed [43] and eventually brought to mechanical failure [44].

The structures encompassed in one voxel include alveoli, gas, vessels, fluids, and interstitial tissues. If we assign to the alveoli the role of loading the inspiratory gas, we should affirm that the computed strain must be attributed to their walls. However, inside one typical CT voxel, there are hundreds of alveoli [45] and their inflation process is not linear. There are sequential processes of recruitment and inflation [46], and moreover, after the alveolus starts its inflation, the amount of strain born by the alveolar walls depends on the morphological alteration of its shape [47] rendering the stress/strain relation of the single groups of alveoli as nonlinear [48]. In fact, the process of sequential recruitment increases the number of available alveoli and make it possible to subdivide the inflation-related strain between newly opened units.

### Distribution pro-inflammatory M1 macrophages using PET

[^68^Ga] Ga-DOTA-TATE is a clinically available radiopharmaceutical binding to SSTR2 and widely used for neuroendocrine tumors imaging. The SSTR are normally expressed on endocrine organs, spleen, thyroid, adrenal, and pituitary glands, while they show little to no expression in healthy lungs.

Among somatostatin receptors, [^68^Ga]Ga-DOTA-TATE has the highest affinity for somatostatin receptors type 2 [18], widely expressed specifically by pro-inflammatory M1 macrophages [17], and for this reason, has already been used successfully to detect macrophagic activation in atherosclerosis-related inflammatory processes [49–51].

The macrophages, both resident in the alveoli and recruited from the bloodstream, have a pivotal role during the initial phase of lung inflammation and ARDS [52, 53]. For this reason, the [^68^Ga]Ga-DOTA-TATE is an ideal candidate for investigating the presence of activated M1 alveolar macrophage during ARDS.

In healthy lungs, long-lived resident alveolar macrophages are the predominant population in the alveoli and are mainly expressing immunosuppressive M2 characteristics. In response to noxious stimuli of the lung, however, circulating monocytes are recruited into the alveolar space, undergo an M1 polarization and thus secrete cytokines and mediators that promote a pro-inflammatory response. This process happens in the early stages of the pathogenesis of ARDS, also known as exudative phase, when mediators produced by M1 macrophages, attract neutrophils from the intravascular space, contributing to the elimination of noxious factors, but also to the tissue damage seen in ARDS. In the later stages of ARDS, after the pathogenic stimuli are eliminated, a gradual shift of resident and recruited macrophages from the M1 to the M2 phenotype occurs. At this stage, M2 macrophages play a key role in mitigating the immune response, producing anti-inflammatory cytokines, and participating in lung tissue repair by phagocytizing debris of necrotic and apoptotic cells [54, 55].

Our group has already tested the efficacy of [^68^Ga]Ga-DOTA-TATE to signal the presence of inflammation during lung injury in a porcine model of ARDS, comparing it with [^18^F]FDG–PET and with histology [19]. It is worth noting that SSTR2 receptors are actively expressed only in M1 pro-inflammatory macrophages [17], while other SSTR subtypes are not upregulated on the M1 polarized macrophages.

This makes the [^68^Ga]Ga-DOTA-TATE highly specific for detecting the presence of activated macrophages and undoubtedly gives it an advantage over the FDG in detecting cell-mediated early inflammation in acute lung injury.

^18^F-FDG is a glucose analog and lacks specificity, although it is acknowledged that ^18^F-FDG may accumulate in activated macrophages [56] than in other subtypes because of the higher glycolytic activity in the pro-inflammatory population. However, it should be mentioned that the increase of glycolytic activity also varies depending on the type of stimulation received by macrophages, with stimulation via the alternative pathway resulting in fewer metabolic effects [57].

Bitker and colleagues have studied lung inflammation by measuring the presence of macrophages using the different PET-tracer named [^11^C]PK11195 which is a ligand of the so-called translocator protein (TSPO). TSPO is a mitochondrial receptor found in macrophages and neutrophils [58]. Although associated with the state of lung inflammation by tissue examination by Bitker [15], this protein is expressed by macrophages in both conditions M1 (proinflammatory) and M2 (anti-inflammatory). Moreover, a recent paper reports that the TSPO is downregulated on pro-inflammatory ‘M1’ human macrophages [59]. This makes [^11^C]PK11195 not genuinely specific for inflammation per se, but surely able to generally signal the presence of macrophages.

### Critical appraisal of the methods

#### The lung injury model

We used a well-established dual-hit VILI model [10, 60, 61]. The advantage of applying in sequence the two-hits injury resides mainly in the steadiness of the alterations in lung mechanics and gas exchange during the course of the experiment [60]. To prevent differences associated with varying mechanical ventilation times, the total ventilation time was kept equal in both animal groups.

*Computation of voxel-associated strain* is surely one of the strengths of the present paper. However, the assessment of strain with such a *high granularity* warrants important reflections. CT scans portray voxels subtending alveoli in a broad range of inflation volumes and not necessarily “functionally connected” to the main airways. In particular, the denominator of Eqs. 2 and 3 can reach very low numbers (i.e. when at FRC the corresponding voxel subtends hypo-inflated or even non-inflated alveoli) and the consequently computed strain reaches very high values. The problem posed by non-ventilated areas in the computation of voxel-associated strain on the lung during mechanical ventilation has been already encountered in other studies. Already in 2010, Caironi and others [62] hypothesized a relation between the potentially recruitable lung and the end-inspiratory alveolar strain, by adjusting the denominator of strain equation for the recruited volumes by assuming their CT density on the basis of preceding observations. The same problem was acknowledged by Paula et al. [63] who adjusted strain calculation using the experimental values deriving from average measurements of regional aeration. However, the use of the recruited volume for adjusting the denominator of the strain equations (see Eqs. 2 and 3) is also prone to generate flaws in strain computation. In our study, we decided not to correct for the recruited volumes and to present the original, untreated data. This has a scientific rationale behind it. There is a general consensus that strain should be computed using as a reference the unstressed volume of lung structures [5]. At the microscopic level an atelectatic structure is certainly in an unstressed state; however, when gas start entering a group of alveoli (that would allow it to be classified as recruited area), it does not guarantee that the equilibrium point between inward and outward forces is reached. In fact, as pointed out by the studies of micromechanics [48], although groups of alveoli can start “loading” gas (and becoming “recruited” by definition), they are still in a process of unfolding their scaffolding and still unable to generate any elastic recoil [64]. This means that the use of the recruited volume for adjusting the denominator of Eqs. 2 and 3 is also by itself prone to generate flaws in strain computation. In contrast, when considering the translational applicability of strain calculation in a clinical context, it is important to recognize that the modification of end-expiratory lung volume through PEEP application can alter the strain exerted on lung structures.

In this context, it is correct to take into account the possibility that animals in the VILI group, ventilated after VILI induction with a tidal volume of 6 ml/kg and PEEP adjusted according to the ARDSnet F_I_O_2_/PEEP table [21], may have experienced an additional strain-related inflammation due to higher levels of PEEP applied during ventilation (see Table 1). However, it is worth noting that several translational observations [65] suggest that high dynamic strain (applied during injurious ventilation in the VILI group) causes more evident damage than the steady application of a high PEEP.

Due to the study design, which involved two defined groups (healthy controls vs. animals exposed to VILI), it was not possible to quantitatively analyze a dose–response relationship between strain and inflammation. This type of analysis would have required a larger sample size and potentially the induction of varying degrees of damage at the beginning of the experiment.

In our model, during VILI induction, the animals were exposed to remarkable strain levels (corresponding to repeated step changes from ZEEP to 40 cmH_2_O) that can be considered very high when compared with the pattern of ventilation customarily used in clinical routine. With the premises deriving from the choice of the model and the computation method described before, the calculation of voxel-associated strain using CT imaging surmounts the value of strain measured at airway opening [6].

#### PET-tracer

The non-specificity of [^18^F]FDG obliges complex calculations of the tracer uptake to assess the contribution of the active use of glucose by the cells involved the inflammation cascade and differentiate from other cells that, although using glucose, are transient bystanders. Instead, for [^68^Ga]Ga-DOTA-TATE, we could apply a plain SUVR calculation. Being in our lung PET slice the activated macrophages and the [^68^Ga]Ga-DOTA-TATE tracer either in the lung structure or in the blood, the SUVR computation [28] allowed us to know the proportion of [^68^Ga]Ga-DOTA-TATE in relation to its concentration inside the heart chambers representing the blood pools (there is no uptake of [^68^Ga]Ga-DOTA-TATE in the myocardium walls which may confound the segmentation of the ventricle [19], as can happen with [^18^F]FDG). We shot a series of PET images per each transverse plane and we chose a timing for their analysis corresponding to 3596 s (≈ 60 min) in line with the available literature and our previous tests [19].

The visualization of macrophages during inflammation can also benefit from the use of tracers other than [^68^Ga]Ga-DOTA-TATE. Apart from the previously discussed [^11^C]PK11195, promising results have also been achieved using markers that target the folate receptor β in studies of myocarditis [66] or rheumatoid arthritis [67]. Studies about the specificity and sensitivity of [^68^Ga]Ga-DOTA-TATE as a diagnostic test for detecting pulmonary inflammation are lacking. More studies have been conducted to assess the diagnostic performance of [^68^Ga]Ga-DOTA-TATE in detecting other kinds of inflammation processes, like atherosclerosis. [^68^Ga]Ga-DOTA-TATE demonstrated a better power than [^18^F]FDG to discriminate high-risk from low-risk coronary atherosclerotic lesions, allowing for a clear interpretation of coronary signals [51]. Even if there are no studies that have directly compared [^68^Ga]Ga-DOTA-TATE and [^18^F]FDG as diagnostic methods to assess pulmonary inflammation, a possible hypothesis that is worth investigating has been enunciated by Li et al. [49]. They affirm that the main difference lies in tracer kinetics. [^68^Ga]Ga-DOTA-TATE binds specifically to SSTR2, and its uptake is limited by the number of saturated SSTR2 receptors. On the other hand, [^18^F]FDG is continuously metabolized by activated macrophages and is more susceptible to nonspecific uptake mechanisms, such as increased vascular permeability due to inflammation. Therefore, theoretically, [^18^F]FDG should be more sensitive than [^68^Ga]Ga-DOTA-TATE, but potentially less specific. As already said, this hypothesis needs to be confirmed by a purposely designed study.

### Shannon’s mutual information

Mutual information measures the amount of information that one variable contains about another or the reduction in uncertainty of one variable when we know the other [68]. Although nowadays extensively used as a tool for image registration [69], mutual information was originally introduced as a similarity measure between images [70]. In particular, the rationale for using it in the present study is that no assumption is needed of a prior functional relationship between the images, it is robust towards outliers, and can be calculated efficiently [68]. The absence of assumptions about the relationship between the image intensities of the two studied modalities makes mutual information highly versatile and powerful without the need for prior segmentation [70].

In its general definition, computation of Shannon’s mutual information yields values that might vary notably in relation to the size and the content of the analyzed images [38]. This may render difficult the comparison of two series of images instead of a couple of them. For this reason, many attempts have been proposed to “normalize” the output of Shannon’s mutual entropy to a fixed range [38] to compare different pools of data and to simplify their interpretation. A mathematical solution to this problem has been published some years ago by Lange and Grubmüller [32] in the field of molecular dynamics, with the scope of avoiding the original range of the output from the original Shannon’s equations [between zero and infinite], which they defined “unfamiliar” and without obvious interpretation. To solve this, they proposed the method of calculation that yields results in the range of [0…1] that we have used in the present paper and allowed the reported comparisons.

#### The shear strain

Estimation of strain from density changes derived from CT does not allow to compute the so-called shear strain (the strain parallel to the surfaces of the voxel), although in terms of energy dissipation, this component can play a non-negligible role.

#### Clinical perspective

It is theoretically reasonable to use a PET tracer in combination with a PET–CT scanner to detect inflammation and diagnose active lung injury in patients admitted to intensive care. The use of PET–CT methods has been increasing in recent years [71], but more research is needed before it can become part of routine clinical practice. It is important to conduct extensive studies to test the specificity and sensitivity of new tracers for each lung inflammatory disease that requires a PET diagnosis. In addition, each new method should undergo a Health Technology Assessment evaluation before its introduction [57].

## Conclusion

Using PET–CT image analysis, we have been able to compute the field of lung strain and to mark the position of activated macrophages using the selective SSTR2 PET-ligand [^68^Ga]Ga-DOTA-TATE. We have analyzed the information provided by these imaging methods first separately and then in a joint fashion, by applying a method based on the theory of information. Macrophages, among the first inflammatory cells to be activated in the place of inflammation, gather in the areas where strain induced by mechanical ventilation is high.

For the first time we show a strong regional coupling between high mechanical strain and cell-mediated inflammation. These findings may have future clinical relevance for both monitoring of VILI-associated inflammation and its potential treatments (e.g., for targeted anti-inflammatory therapies of ARDS).

## Data Availability

The data set analyzed during the current study are available from the corresponding author on reasonable request.

## Abbreviations

[^18^F]FDG: 2-Deoxy-2-[^18^F]fluoro-d-glucose
ABG: Arterial blood gas
*c*_lung_: Radioactivity concentration in voxel belonging to the lung
*c*_ref_: Radioactivity concentration in a reference group of voxels
CT: Computed tomography
Δ*P*: Driving pressure
ETT: Endotracheal tube
F_I_O_2_: Fraction of inspired oxygen
FOS: Field of strain
HU: Hounsfield units
IC: Inspiratory capacity
IHM: Inspiratory hold maneuver
MBq: Mega Becquerel
Mid-T: Computed tomography slice at mid-thoracic levels
Para-D: Computed tomography slice at para-diaphragmatic level
PEEP: Positive end-expiratory pressure
*P*_es_: Esophageal pressure
*P*_TP_: Transpulmonary pressure
PET: Positron emission tomography
PET–CT: Combined positron emission tomography and computed tomography
*P*_ga_: Gastric pressure
[^11^C]PK11195: [*N*-Methyl-^11^C]-1-(2-chlorophenyl)-*N*-(1-methylpropyl)-3-isoquinoline-carboxamide
RM: Recruitment maneuver
RR: Respiratory rate
SpO_2_: Peripheral oxygen saturation
SSTR2: Somatostatin receptor subtype 2
SUVR: Standardized uptake value ratios
*V*_FRC_: Lung volume at functional residual capacity
VILI: Ventilator-induced lung injury
VoStrain: Voxel-associated normal strain
*V*_T_: Tidal volume
*V*_vox_,_gas_: Volume of gas contained in a voxel
*V*_vox_: Volume of one voxel
ZEEP: Zero end-expiratory pressure
